# How do spherical bacteria regulate cell division?

**DOI:** 10.1042/BST20240956

**Published:** 2025-04-17

**Authors:** Félix Ramos-León, Kumaran S. Ramamurthi

**Affiliations:** Laboratory of Molecular Biology, National Cancer Institute, National Institutes of Health, Bethesda, MD 20892, U.S.A.

**Keywords:** *Bacillus subtilis*, *Deinococcus*, *E. coli*, MinCDE, Min system, *Neisseria*, Synechococcus, Cyanobacteria

## Abstract

Many bacteria divide by binary fission, producing two identical daughter cells, which requires proper placement of the division machinery at mid-cell. Spherical bacteria (cocci) face unique challenges due to their lack of natural polarity. In this review, we compile current knowledge on how cocci regulate cell division, how they select the proper division plane, and ensure accurate Z-ring positioning at mid-cell. While *Streptococcus pneumoniae* and *Staphylococcus aureus* are the most well-studied models for cell division in cocci, we also cover other less-characterized cocci across different bacterial groups and discuss the conservation of known Z-ring positioning mechanisms in these understudied bacteria.

## Introduction

**Figure 1:**
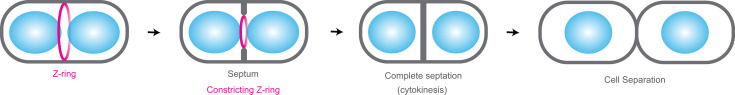
Cell division in rod-shaped bacteria. After chromosome (blue) duplication, FtsZ (magenta) polymerizes at mid-cell, forming the Z-ring. The division machinery is then recruited to the site, catalyzing the synthesis of the septal cell wall (gray). As the septum forms, the Z-ring constricts. Once septation is complete, cytokinesis is achieved as it separates the cell into two distinct compartments. The action of cell wall hydrolases and then facilitates the final splitting, producing two identical daughter cells.

Cell division is an essential process for all living organisms. Many bacteria accomplish this through a process known as binary fission, wherein a parent cell asexually splits into two identical daughter cells. During this process, the parental cell positions the cell division machinery, called the divisome, at the cell division site. This protein complex builds a new cell wall in the middle of the cell, called the division septum. As this new cell wall forms, it eventually splits the cell into two separate daughter cells, thereby completing the process known as cytokinesis. The first step in bacterial cell division is the localization of FtsZ, a nearly universally conserved bacterial homolog of tubulin, which polymerizes in a GTP-dependent manner into short discontinuous filaments that form a ring-like structure termed the ‘Z-ring’ at the cell division site. These short filaments exhibit a property termed ‘treadmilling’, wherein continuous FtsZ polymerization on the one end and depolymerization at the other end give the appearance that the short polymer is moving, despite individual FtsZ monomers in the middle of the polymer remain stationary [1,2]. The polymeric Z-ring recruits the downstream components of the divisome, initiating the process of septation [3] (Figure 1).

A critical step in this process is the proper positioning of the Z-ring at the next plane of cell division, often but not exclusively at approximately mid-cell to yield two similarly sized daughter cells. To ensure proper Z-ring placement, bacteria have evolved several mechanisms. The best characterized of these is the so-called ‘Min’ system in the rod-shaped Gram-negative bacterium *Escherichia coli* that is partially conserved in the rod-shaped Gram-positive bacterium *Bacillus subtilis* [4,5]. In *E. coli*, the Min system consists of three proteins: MinC, MinD, and MinE. MinC interacts with FtsZ and inhibits FtsZ polymerization, while the MinDE complex contributes to the localization of MinC near the poles, away from the mid-cell. This system exhibits an oscillatory behavior, wherein the MinCDE complex dynamically relocates *en masse* from one pole to the other. The result is a time-averaged lower cellular concentration of the FtsZ-inhibitory Min proteins near mid-cell, and a higher concentration near the poles, to promote FtsZ positioning near mid-cell [6]. At the same time, other cell division components that interact directly with FtsZ contribute to stabilization and regulation of FtsZ polymerization in the Z-ring [7]. This oscillatory process relies on the ATPase activity of MinD. Membrane-bound MinD recruits MinE, which stimulates hydrolysis of ATP by MinD, leading to the detachment of the MinDE complex from the membrane [8]. However, the Min system is not universally conserved in bacteria, and even when components of the Min system are conserved, they may act differently than they do in *E. coli*. In *B. subtilis*, for example, the MinCD complex is recruited by MinJ, which is recruited by the scaffold protein DivIVA [9,10]. DivIVA localizes to negatively curved membranes at the cell poles and invaginating membranes at division sites [11,12]. Unlike in *E. coli*, the *B. subtilis* Min system does not exhibit oscillatory behavior from pole to pole and instead largely localizes to new cell division sites to prevent aberrant cell division near mid-cell [9,10,13,14]. Nonetheless, a recent report showed that *B. subtilis* MinD also exists in a dynamic state between cytosol and membrane, suggesting the presence of a mechanism to stimulate MinD ATPase activity in this organism as well [15].

A second well-studied mechanism for cell division site selection, independent of the Min system, is ‘nucleoid occlusion’, whereby Z-ring formation is largely prevented over a chromosome to ensure that the chromosome is not bisected during cytokinesis. In *E. coli*, nucleoid occlusion is mediated by the protein SlmA [16]. SlmA, a member of the TetR family, binds to palindromic sequences known as SlmA-binding sites (SBS), which are distributed throughout the *E. coli* chromosome, except in the *ter* region (which in *E*. coli is located close to the plane of cell division) [17,18]. SlmA binds DNA as a dimer-of-dimers, enabling direct interaction with the FtsZ C-terminal domain [19], which promotes the disassembly of FtsZ filaments into shorter species without affecting its GTPase activity [20]. Some studies have demonstrated that the FtsZ-SlmA-SBS complex can form dynamic, crowding-induced condensates, suggesting that phase separation may play a role in spatiotemporal organization of cell division [21]. In *B. subtilis*, nucleoid occlusion is regulated by Noc, a ParB-like protein [22,23]. Noc binds to 16 bp DNA sequences (termed ‘Noc-binding sites’) that are dispersed throughout the chromosome but, like SBS in *E. coli*, are largely absent near the *ter* region [24]. Noc also binds to the cytoplasmic membrane through an N-terminal amphipathic helix [25]. Recent work has revealed that Noc function is regulated by binding to the nucleotide CTP [26,27]. When Noc is unbound to DNA, its amphipathic helix adopts an autoinhibitory conformation that prevents membrane binding. Upon binding DNA, the affinity of Noc for CTP is stimulated, which triggers a conformational change that frees the amphipathic helix and permits interaction with the plasma membrane while Noc spreads across the chromosome. CTP hydrolysis then leads to the dissociation of Noc from the DNA, resetting the protein for another binding cycle. Unlike SlmA, Noc does not appear to bind directly to FtsZ. Instead, it is hypothesized to regulate Z-ring placement by preventing divisome assembly at the membrane sites where it localizes.

Both the Min system and the nucleoid occlusion have been studied chiefly in rod-shaped model organisms [28]. The rod shape of *E. coli* and *B. subtilis*, for example, offers an advantage in providing a self-evident mid-cell position for the Z-ring, as the presence of poles creates a natural polarity. However, different bacteria exhibit a variety of shapes [29], which can introduce unique challenges in regulating binary fission. A distinct case is that of coccus-shaped bacteria, which are spherical and, therefore, lack the inherent polarity found in rod-shaped bacteria and can, therefore, theoretically divide in an infinite number of division planes [30]. Cocci are present across most bacterial groups, suggesting that spherical shape has evolved from a rod shape multiple times throughout evolution. These shape transitions often involved the loss of machinery essential for lateral cell wall synthesis, such as the actin homolog MreB, which is critical for elongation of rod-shaped cells [31]. This diversity implies that cell division regulation and Z-ring positioning systems co-evolved with changes in cell shape, ensuring accurate and precise binary fission. Due to the unique challenges cocci face during division, they probably encode specific mechanisms that have evolved to compensate for their lack of polarity or to generate geometric cues that facilitate the correct selection of the division plane. Interestingly, many of the cell division mechanisms characterized in spherical bacteria that we will discuss in this review nonetheless depend on morphological changes that result in a slight polarization of the cell, which may be either permanent, as seen in ovococci, or temporary as a part of the normal cell cycle. In this review, we will summarize the current understanding of the mechanisms controlling the early stages of cell division in various cocci from different groups, with a focus on those involved in Z-ring positioning. Many components of the cell division machinery in cocci have been identified through their homology to conserved elements in rod-shaped relatives such as *E. coli* and *B. subtilis*. Our focus will be on cell division components that are specific to cocci that distinguish them from rod-shaped bacteria, while also discussing more broadly conserved cell division proteins that play slightly different roles in cocci.

### Mechanisms for Z-ring positioning in Gram-positive cocci

The phylum Bacillota (formerly Firmicutes) includes many of the most well-known Gram-positive cocci, including important pathogens such as *Streptococcus pneumoniae* and *Staphylococcus aureus*. These bacteria have served as key models for studying the regulation of cell division in Gram-positive cocci, uncovering unique mechanisms that differ from those found in rod-shaped bacteria.

#### S. pneumoniae

*S. pneumoniae* is a human commensal bacterium capable of causing opportunistic infections in the respiratory tract. *S. pneumoniae* cells are ellipsoidal in shape and divide along consecutive parallel planes, resulting in the formation of characteristic chains of cells. In *S. pneumoniae*, FtsZ orchestrates the recruitment of the cell wall synthesis machinery to mid-cell, where peptidoglycan synthesis occurs through two distinct modes: septal (to form the division septum) and peripheral (which elongates the cell). Septal cell wall synthesis, co-ordinated by the divisome, is carried out by the class B penicillin binding protein (PBP) PBP2x/FtsW complex that moves circumferentially at mid-cell, driven by peptidoglycan synthesis, resulting in the separation of the dividing cells [32–35]. Peripheral cell wall synthesis is orchestrated by the elongasome, with the class B PBP2b/RodA complex playing a key role. This complex is recruited to mid-cell and extends outward to drive cell elongation [32,33]. Recent studies have shown that the elongasome complex migrates circumferentially at mid-cell, driven by peptidoglycan synthesis. However, outside the mid-cell zone, elongasome components exhibit more diffuse movement, spreading across the entire cell surface [36]. In addition to class B PBPs, *S. pneumoniae* possesses three class A PBPs (bifunctional enzymes with both glycosyltransferase and transpeptidase activities), designated PBP1a, PBP1b, and PBP2a [37]. Class A PBPs are generally thought to play a crucial role in the maturation of the peptidoglycan synthesized by both the elongasome and the divisome and also probably participate in peptidoglycan repair. PBP1a is involved in cell elongation and exhibits dynamics similar to those of elongasome components [36]. PBP1a also forms a synthetic lethal pair with PBP2a [38], and the activity of PBP2a is regulated by a pneumococcal-specific protein termed MacP [39]. The role of PBP1b remains elusive.

**Figure 2:**
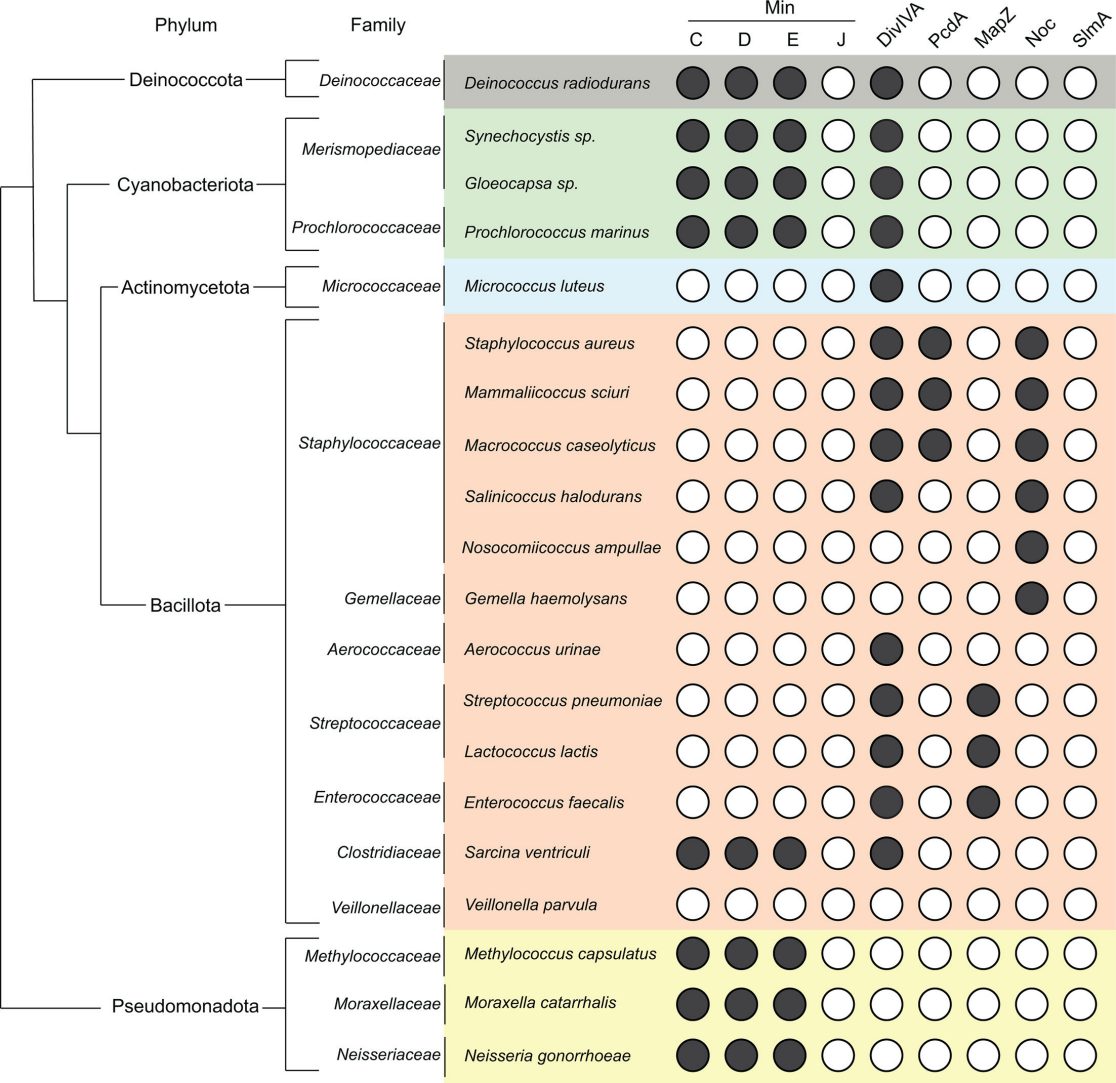
Presence of Z-ring positioning systems in coccoid representatives from different bacterial groups. Presence (black circles) or absence (white circles) was determined by BLAST searches using the indicated gene from *Bacillus subtilis*, *Escherichia coli*, *Staphylococcus aureus,* or *Streptococcus pneumonia*. Phyla are indicated to the left. NCBI GenBank IDs: *Deinococcus radiodurans* R1 (GCA_021378295.1), *Synechocystis* sp. PCC 6803 (GCA_000009725.1), *Gloeocapsa* sp. PCC 7428 (GCA_000317555.1), *Prochlorococcus marinus* MIT9313 (GCA_000011485.1), *Micrococcus luteus* NCTC 2665 (GCA_000023205.1), *Staphylococcus aureus* USA300_FPR3757 (GCA_000013465), *Mammaliicoccus sciuri* FDAARGOS_285 (GCA_002209165.2), *Macrococcus caseolyticus* JCSC5402 (GCA_000010585.1), *Salinicoccus halodurans* H3B36 (GCA_001005905.1), *Nosocomiicoccus ampullae* DSM 19163 (GCA_019357495.1), *Gemella haemolysans* NCTC 10459 (GCA_900638055.1), *Aerococcus urinae* CCUG 36881 (GCA_001543175.1), *Streptococcus pneumoniae* sv. 2 D39 (GCA_000014365), *Lactococcus lactis lactis* IL1403 (GCA_003722275.1), *Enterococcus faecalis* Portland, ATCC 29212 (GCA_000742975.1), *Sarcina ventriculi* NCTC 12966 (GCA_900456775.1), *Veillonella parvula* DSM 2008 (GCA_000024945.1), *Methylococcus capsulatus* Bath (GCA_000008325), *Moraxella catarrhalis* BBH18 (GCA_000092265.1), and *Neisseria gonorrhoeae* FA 1090 (GCA_000006845.1). Note that ‘DivIVA’ in Cyanobacteriota refers to the paralog protein Cdv3. Refer to Supplementary Table S1 for percentage identity and locus tags of the proteins indicated in this figure.

**Figure 3:**
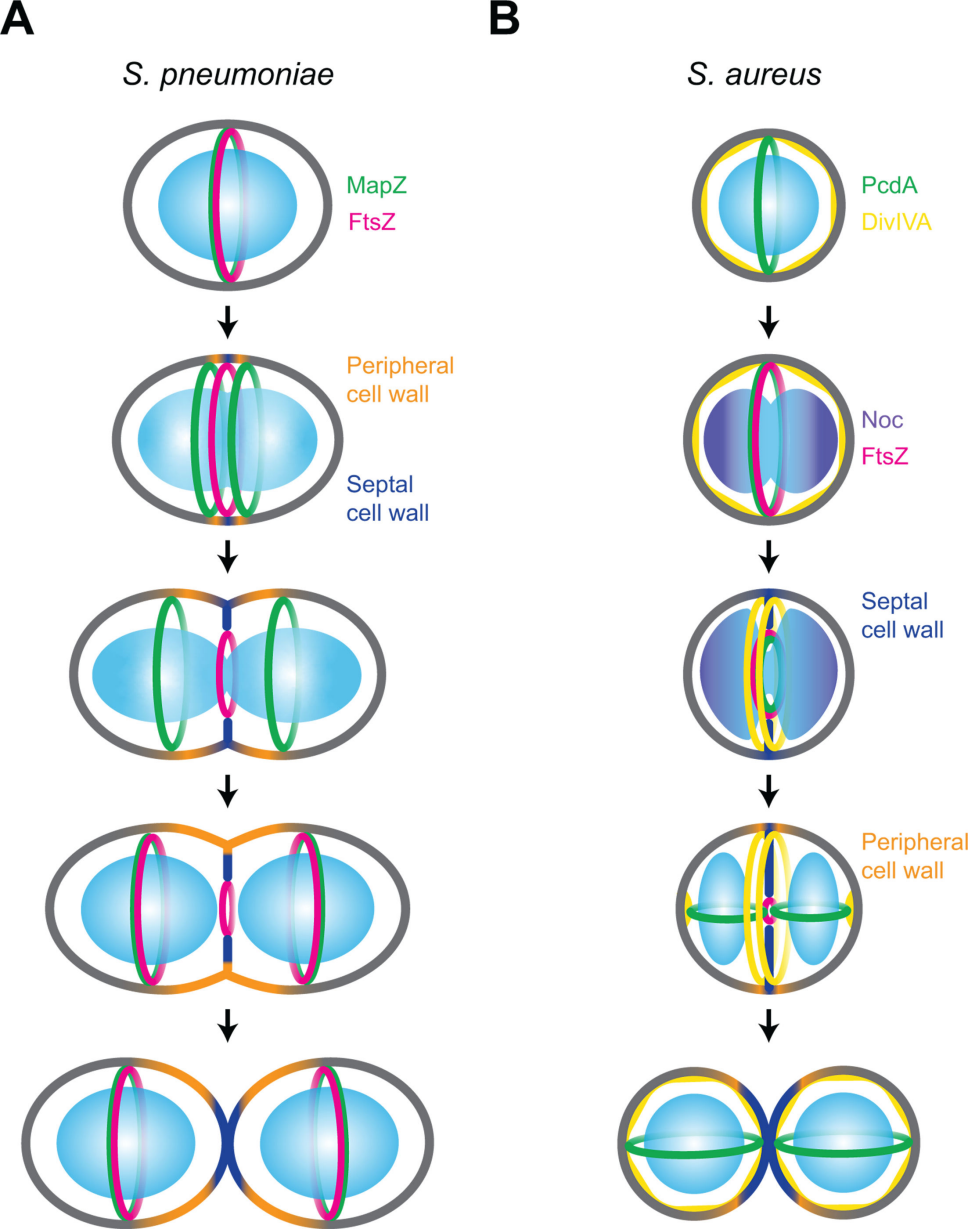
Molecular mechanisms for Z-ring positioning in Gram-positive cocci. (**A**) Z-ring positioning in *Streptococcus pneumoniae*. In *S. pneumoniae*, MapZ (green) forms a ring at the mid-cell and directly interacts with FtsZ (magenta), recruiting it to form the Z-ring over the chromosome. As DNA replication occurs and the chromosomes start segregating toward the poles, peripheral (orange), and septal (dark blue) peptidoglycan synthesis take place simultaneously. During this process, the MapZ ring splits in two and migrates toward the future daughter cell hemispheres. Peripheral cell wall synthesis continues contributing to MapZ ring movement, while septal peptidoglycan synthesis and Z-ring constriction proceed in co-ordination with chromosome segregation. In the daughter cell hemispheres, MapZ recruits FtsZ in preparation for the next round of cell division. Upon cell separation, the resulting daughter cells inherit a predetermined division site marked by the MapZ-FtsZ complex. Cell periphery depicted in gray; nucleoid depicted in blue. (**B**) Z-ring positioning in *Staphylococcus aureus*. In pre-septated spherical cells, PcdA (green) forms a ring at mid-cell over the chromosome, while DivIVA (yellow) localizes at the cell periphery. As chromosome duplication and segregation begin, Noc (purple) binds to the origin regions of the chromosomes, preventing FtsZ polymerization near these areas. PcdA recruits FtsZ (magenta) to mid-cell through direct interaction. During septation, DivIVA coalesces at the division site, likely forming two rings on either side of the developing septum (dark blue). Localized periseptal peptidoglycan synthesis (orange) contributes to subtle cell elongation, leading to the formation of temporary poles where a subpopulation of DivIVA is recruited. ‘Polar’ DivIVA recruits PcdA, which redeploys from the constricting septum to form two new rings in each daughter cell that flank the constricting septum roughly perpendicularly. Following cell separation, each daughter cell contains a PcdA ring in a plane roughly orthogonal to the previous division site.

*S. pneumoniae* lacks both the Min system and Noc, which are otherwise conserved in rod-shaped Bacillota (Figure 2). Instead, it uses a distinct mechanism for cell division placement, mediated by the protein MapZ [40,41]. In early stages of the cell cycle, MapZ forms a ring at mid-cell, acting as an anchor that guides the treadmilling of FtsZ and positively regulates Z-ring positioning. Prior to the onset of constriction, the MapZ ring splits into two rings, which gradually move apart as peptidoglycan synthesis elongates the cell and builds the new cell hemispheres (Figure 3A). It is hypothesized that peptidoglycan assembly drives the separation of the MapZ rings toward the daughter cell hemispheres, marking the future division sites. MapZ interacts directly with FtsZ via its cytoplasmic domain and with the cell wall via its extracellular domain [42,43], ensuring proper FtsZ filament treadmilling throughout the division cycle from the septum to the equator of the daughter cells [40,42,43]. The cellular cues that MapZ recognizes to find mid-cell in *S. pneumoniae* are not known. However, recent work in *Streptococcus mutans* may provide a clue [44]. In *S. mutans*, the peptidoglycan is adorned with serotype C carbohydrates (SCCs), which consist of a rhamnose backbone decorated with modifications. At the equatorial rings and cell poles, newly synthesized SCCs are present without these modifications. The absence of proper modifications impairs the recruitment of MapZ to the equatorial rings, highlighting the role of immature SCCs as landmarks for guiding MapZ to the equator of future daughter cells during division. In *S. pneumoniae*, the cell wall is decorated with wall teichoic acids (WTAs) instead. Importantly, variations of WTA levels have been shown to influence cell elongation, and WTA has been shown to be mainly present in areas of active peptidoglycan synthesis [45]. This suggests that the presence of lipoteichoic acid and WTA could influence the division site positioning in *S. pneumoniae*, similar to SCC playing a role in the recruitment of MapZ in *S. mutants*.

Phosphorylation of some cell division proteins by the serine/threonine protein kinase StkP also plays an important role in controlling cell division in *S. pneumoniae* [46]. Although MapZ is an StkP substrate, the phosphorylation of MapZ does not appear to affect its interaction with FtsZ [40,43] or the Gram-positive-specific cell division factor DivIVA [40,47]. In *S. pneumoniae*, DivIVA localizes to the cell poles and as a double ring at the division site [48]. Deletion of *divIVA* leads to defects in cell elongation by contributing to the co-ordination of peripheral peptidoglycan synthesis and septum splitting [48–50]. Additionally, StkP phosphorylates DivIVA, and this modification is essential for normal cell division [51]. Moreover, recent research in *Streptococcus suis*, a pig pathogen, revealed that expressing a phosphomimetic form of DivIVA (a single amino acid DivIVA variant that mimics phosphorylated DivIVA) disrupts the septal localization of the peptidoglycan hydrolase MltG, resulting in cells that are shorter than wildtype [52]. This suggests that the phosphorylation of DivIVA may play a role in regulating the timing and co-ordination of peripheral peptidoglycan synthesis with cell division.

Two more mechanisms have been implicated in cell division site selection in *S. pneumoniae*. First, the co-ordination of cell division with chromosome replication and segregation is crucial for accurate division site selection. This process is mediated by CcrZ, a protein conserved in Bacillota that regulates the activity of the DNA replication initiator DnaA. CcrZ directly interacts with FtsZ and localizes at mid-cell, where it activates DnaA-dependent replication initiation. CcrZ is conserved in other Bacillota and also appears to co-ordinate chromosome segregation in *S. aureus*. However, the mechanism may differ, as the *S. aureus* CcrZ protein cannot complement *ccrZ* depletion in *S. pneumoniae* [53,54]. Second, many *Streptococcus* strains produce a polysaccharide capsule that is synthesized exclusively at the division septum [55], which requires the *Streptococcus*-specific protein RocS to co-ordinate capsule production with the cell cycle. RocS interacts with FtsZ and the chromosome segregation protein ParB, while simultaneously interacting with the tyrosine kinase CpsD, which is essential for capsule production. This interaction network ensures that capsule synthesis is tightly linked to cell division and chromosome segregation [56].

Outside of the genus *Streptococcus*, the regulation of cell division in the order *Lactobacillales* has not been extensively studied. However, while MapZ is conserved in representative species of the genera *Lactococcus* and *Enterococcus*, it is absent in other genera within the class *Lactobacilliales*, such as *Aerococcus* (Figure 2) [57]. Interestingly, unlike the ovoid streptococci, species of *Aerococcus* are more spherical in shape and grow in pairs, tetrads, or clusters [58], suggesting that MapZ-dependent site selection may be a defining characteristic of ovoid bacteria. The presence of peripheral cell wall synthesis within the genus *Aerococcus* has not been studied, and the mode of growth of these organisms remains unknown. Moreover, some research has shown that, in *Lactococcus lactis*, an imbalance in the two cell wall synthesis machineries can drive a coccus-to-rod transition during growth under certain conditions [59]. Thus, some ovoid cells may exhibit a more flexible growth pattern than previously thought, which may necessitate unique strategies to properly mark cell division sites.

#### S. aureus

*S. aureus* is a Gram-positive, spherical bacterium that is commonly a part of the human microbiota but can also act as an opportunistic pathogen. Although theoretically a spherical cell may divide along an infinite number of different planes, *S. aureus* cells do not randomly choose a division plane. Instead, *S. aureus* cells divide in consecutive orthogonal planes, meaning that each daughter cell undergoes cell division in a plane roughly perpendicular to the division plane of the parental cell [60,61]. This cell division pattern drives the characteristic grape cluster-like growth pattern of *S. aureus*. As described above, cell division in *S. aureus* initiates with the polymerization of FtsZ at mid-cell, which recruits the machinery responsible for septal peptidoglycan synthesis. The first peptidoglycan biosynthetic enzyme recruited to the septum is the class A bifunctional enzyme PBP2 where it synthesizes the initial peptidoglycan layer, known as the ‘piecrust’. Next, the class B PBP/SEDS pair PBP1/FtsW constructs the remainder of the septal peptidoglycan [62,63]. Interestingly, inward septum synthesis is driven not by FtsZ treadmilling but by peptidoglycan synthesis [64,65]. Once septation is complete, the parental cell splits into two daughter cells. This splitting process is very fast (within milliseconds) and requires the actions of cell wall hydrolases [66].

Although *S. aureus* cells were historically considered perfectly spherical throughout the cell cycle, careful microscopy measurements revealed that *S. aureus* slightly elongates during septation [67]. This elongation depends on the class B PBP/SEDS pair PBP3/RodA, which is recruited to mid-cell after PBP1/FtsW, after which they remain localized at the base of the septum, thereby promoting the insertion of sidewall peptidoglycan in a mechanism that is reminiscent of the peripheral cell wall synthesis observed in ovoid bacteria described above [68]. Thus, although *S. aureus* may have been considered as a simpler bacterial system to study cell division due to its presumed restriction of cell wall synthesis to the septum, more recent findings suggest a model of growth that is more similar to that of ovoid bacteria, while displaying much more subtle morphological changes. This revised view, therefore, implies a co-ordination between two different peptidoglycan synthesis machineries. Recent reports have suggested that GpsB, a paralog of DivIVA, may be required for cell elongation. Deleting *gpsB* resulted in cells that became more spherical, similar to the phenotypes arising from deleting *pbp3* or *rodA* [69,70]. Additionally, GpsB bundles FtsZ filaments and stimulates the GTPase activity of FtsZ [71]. GpsB also engages with the machinery responsible for WTA synthesis [72], as well as the class C penicillin-binding protein PBP4 [73]. In sum, these observations suggest that GpsB may orchestrate the synthesis of septal and sidewall peptidoglycan, although how this is mechanistically achieved remains still largely unclear. Interestingly, GpsB has also been shown to play a role in co-ordinating cell division in *S. pneumoniae*, but in this case, deletion of *gpsB* results in elongated cells [26]. Another recently identified cell division component is FacZ, which is conserved in *B. subtilis* and other members of the Bacillota phylum [74]. Deleting *facZ* resulted in abnormal membrane and peptidoglycan accumulations and improper localization of FtsZ. FacZ localizes to the peripheral membrane and is enriched at the periseptal region (at the base of the septum, where it meets the cell periphery), where it may antagonize GpsB in the stabilization of Z-rings during the onset of cell division via an as-yet undetermined mechanism.

The historical focus on *S. aureus* as a model for understanding peptidoglycan assembly [75] led to an early hypothesis that the cell wall may provide epigenetic information that could dictate where the subsequent cell division plane may occur [76]. Studies using atomic force microscopy revealed that the nascent cell division septa formed a belt of peptidoglycan that the authors referred to as displaying a ‘piecrust’-like texture [76]. Interestingly, remnants of this piecrust remained in the daughter cell and happened to be roughly orthogonal to the future cell division plane. The authors, therefore, proposed that this intrinsic feature of dividing a coccus may influence the selection of the subsequent division plane. Indeed, differently isolated mutants of *E. coli* that grew as spherical cells instead of as rods reportedly displayed a propensity to divide in sequentially orthogonal division planes [77,78], suggesting that orthogonal cell division may spontaneously arise in spherical cells without the need for any specialized mechanisms. However, the notion that a peptidoglycan scar can dictate the relative angle of the next cell division plane was questioned by Monteiro et al. who employed fluorescence microscopy and scanning electron microscopy to demonstrate that when the flat division septum transformed into a curved surface after cell splitting, the resulting surface represents less than one hemisphere of each daughter cell [67]. Thus, unlike what was previously assumed, any peptidoglycan scar that was generated from cell division would not divide the spherical cell perfectly in half and thus may not be an appropriate cellular landmark to orient the next cell division plane, thereby reigniting the possibility that more active (potentially species-specific) mechanisms may exist that guide division plane selection in cocci.

*S. aureus* lacks the Min system, but it does harbor the nucleoid occlusion protein Noc (Figure 2). Deleting *noc* leads to Z-ring formation over the chromosome, resulting in DNA breaks [79]. Complicating matters further, in *S. aureus*, Noc also influences DNA replication initiation, since deleting *noc* in *S. aureus*, but not in *B. subtilis*, results in over-replication of the chromosome [80]. Noc binds to sequences near the origin of replication in the *S. aureus* chromosome and somehow influences the ability of DnaA to initiate replication. This dual function of Noc in nucleoid occlusion and replication control suggests a tight coupling between division and DNA replication. However, in the absence of Noc, many cells continue to divide normally, suggesting the existence of additional Z-ring placement factors.

One such additional system is the recently identified early cell division protein PcdA, an McrB family AAA^+^ NTPase that is specifically involved in orthogonal plane selection in *S. aureus* [81]. Deleting *pcdA* resulted in cells dividing in planes that were not orthogonal to the previous cell division plane. Deleting both *pcdA* and *noc* resulted in an additive impairment of cell division, suggesting that both proteins operate via independent pathways. PcdA co-constricts with FtsZ during cell division, but before cytokinesis is completed, a subpopulation of PcdA redeploys to the future (orthogonal) cell division site and arrives to that site before FtsZ [81]. In the absence of PcdA, FtsZ fails to redeploy efficiently to the next cell division plane, consistent with the notion that PcdA directs FtsZ to the next cell division plane. The dynamic localization of PcdA seems to depend on its nucleotide binding state. Biochemical studies revealed that PcdA interaction with unpolymerized FtsZ requires ATP binding, whereas PcdA interaction with polymerized FtsZ is nucleotide independent. PcdA localizes to future division sites by binding to *S. aureus* DivIVA. In the pre-divisional cell, DivIVA localizes indiscriminately to the membrane periphery (Figure 3B, first panel; DivIVA represented in yellow). As cell division initiates, DivIVA localizes to the division septum (likely as double rings on either side of the constricting septum, similar to the localization pattern of DivIVA in *B. subtilis*; Figure 3B, third panel) [14]. However, as cell division proceeds and the cell elongates slightly, a subpopulation of DivIVA deploys to the future division sites before septation has concluded (Figure 3B, fourth panel) [81,82]. How does DivIVA recognize the future division plane? One model hypothesized that the subtle change in cell architecture, which causes a micron-scale change in global membrane curvature during cell elongation, may drive *S. aureus* DivIVA localization to the resulting ‘poles’ of the slightly ellipsoidal cell, similar to DivIVA action in *B. subtilis*. This subpopulation of DivIVA serves as a landmark to recruit a subpopulation of PcdA from the constricting septum to the future cell division plane. Consistent with this hypothesis, *rodA* or *pbp3* deletions, which prevent cell elongation, also caused defects in orthogonal cell division plane selection, similar to the deletion of *pcdA*. Moreover, the deletion of *divIVA* also abrogated orthogonal cell division. Thus, cell division in *S. aureus* appears conceptually closer to cell division in *S. pneumoniae*, where a positive regulator controls Z-ring placement, and the machinery involved in cell elongation contributes to morphological changes that signal cell division site selection (Figure 3). Thus, changes in local membrane curvature could be a geometric cue used by *S. aureus* to select the next plane of cell division, which depends on the subtle elongation of the cell during the cell cycle and perhaps also on physical properties of the cell wall that allow deformation at the future division sites.

Given the unique challenges of orthogonal cell division, it is perhaps not surprising that PcdA is limited to Staphylococci and closely related genera such as *Mammaliicoccus* and *Macrococcus* but is absent in other cocci from the order Bacillales such as *Salinicoccus*, *Nosocomiicoccus*, and *Gemella* (Figure 2). Interestingly, *N. ampullae* and *G. haemolysans* lack orthologs of both DivIVA and PcdA, suggesting either that they may rely solely on the nucleoid occlusion system for cell division plane selection or harbor another uncharacterized system involved in this process. Surprisingly, some cocci within the Bacillota phylum, such as *Sarcina ventriculi* (also known as *Clostridium ventriculi*, from the class Clostridia), possess the complete Min system and DivIVA. Members of the genus *Sarcina* grow as tetrads of spherical cells, suggesting a unique, largely unexplored, mode of cell division [83]. In contrast, the coccus *Veillonella parvula*, a unique diderm member within the Bacillota, does not harbor homologs of any characterized system, suggesting yet another distinct mechanism for Z-ring placement.

Outside of the Bacillota, the phylum Actinomycetota (also known as Actinobacteria) also includes spherical Gram-positive members, such as the relatively well-studied *Micrococcus luteus. M. luteus* lacks known mechanisms for Z-ring placement but encodes a DivIVA homolog (Figure 2). In other actinobacteria, DivIVA directs cell wall synthesis at the poles contributing to control of polar growth [84], but the role of DivIVA in *M. luteus* cell biology is not known. Notably, positive regulators of Z-ring positioning have been identified in the filamentous actinobacterium *Streptomyces* [85], but these proteins are absent in *M. luteus*. However, *M. luteus* does encode a homolog of SepH, a regulator of Z-ring formation in filamentous and unicellular actinobacteria [86], although its potential role in controlling cell division in spherical actinobacteria has yet to be explored.

### Mechanisms for Z-ring positioning in Gram-negative cocci

#### Cyanobacteria

The spherical shape is also found in bacterial groups that possess a diderm cell envelope, consisting of a plasma (inner) membrane, an outer membrane, and a thin peptidoglycan layer in the periplasm between the two membranes. These groups have homologs of the Min system, suggesting that Z-ring positioning may be controlled by negative regulators (Figure 2). Among these, the phylum Cyanobacteriota (commonly known as cyanobacteria) includes numerous coccoid-shaped members but tend to encode homologs of the Min system regardless of their morphology [87]. Cyanobacteria also encode Cdv3, a protein that displays low but significant similarity to the Gram-positive DivIVA discussed above, that plays a crucial role in cell division across multiple cyanobacterial models [88]. The genus *Synechocystis* represents the best-characterized coccoid cyanobacterium. In *Synechocystis*, although the Min system is not essential, its deletion nonetheless results in aberrant cell morphology and the formation of minicells, suggesting a role in regulating cell division and Z-ring placement [89]. In contrast, Cdv3 is essential for *Synechocystis* survival, and its depletion leads to the formation of giant spherical cells. In Cdv3-depleted strains, FtsZ forms abnormal rings, suggesting that Cdv3 plays a critical role in Z-ring positioning [90]. In the cyanobacterium *Synechococcus elongatus*, despite the presence of internal thylakoid membranes, the Min system exhibits an oscillatory behavior similar to that observed in *E. coli* [91]. This research also demonstrated that Cdv3 localizes at the septum, facilitating the recruitment of a population of MinC to mid-cell. This suggests that the Min system in cyanobacteria may have a dual role in Z-ring regulation, requiring tight co-ordination between MinE- and Cdv3-dependent mechanisms [91].

#### Deinococcus radiodurans

**Figure 4:**
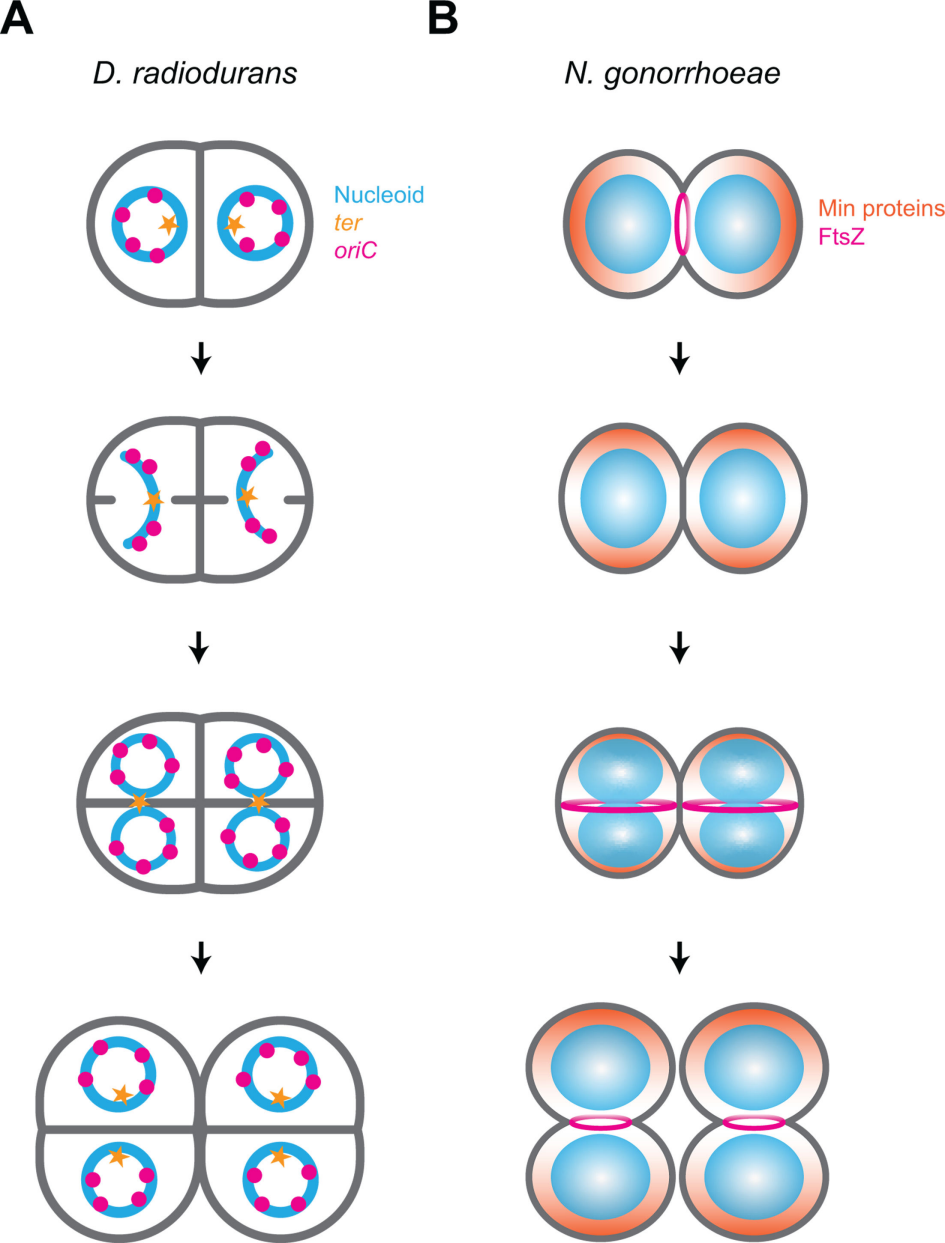
Molecular mechanisms for cell division and Z-ring positioning in Gram-negative cocci. (**A**) Model for cell division and chromosome segregation in *Deinococcus radiodurans*. In *D. radiodurans*, the nucleoid (blue) adopts a toroidal shape, with the *ter* sites (orange star) positioned centrally and the *oriC* sites (magenta circles) encircling the *ter* regions. Septation follows a closing door mechanism, with chromosomes segregating along the long axis of the future daughter cells while the *ter* regions remain at the cell’s center. As the cells approach full septation, the nucleoids take on a circular conformation on either side of the septum, with the *ter* sites clustering at the center of the new septum. Once septation is complete, the nucleoid returns to a toroidal structure, with the *ter* regions centrally located and surrounded by the *oriC* sites. (**B**) Model for regulation of cell division site selection in *N. gonorrhoeae*. The Min system (orange) regulates the positioning of the FtsZ ring (magenta) by forming an oscillating gradient along the long axis of the diplococcus. As septation progresses and cell division nears completion, a new Min gradient is set up along the long axis of the future daughter cells. Chromosome replication and segregation occur along this axis as the FtsZ ring assembles in a plane perpendicular to the newly formed septum. By establishing the Min gradient along the long axis before the parental cell splits into two daughter cells, the cell creates a temporary polarity that helps define the long axis in the daughter cells.

Similar to cyanobacteria, *Deinococcus radiodurans* harbors both the Min system and a DivIVA homolog (Figure 2). Recent studies classify *D. radiodurans* as classical diderm bacterium, indicating the presence of a complex cell envelope that includes an inner membrane, an outer membrane, and an S-layer [92]. However, the exact composition of the S-layer and the presence of a definitive outer membrane remain subjects of debate, with variations depending on the strain examined and the methodologies employed [93,94]. Despite the details of its cell envelope, *D. radiodurans* appears as a group of four cells to form tetrads, and cells divide alternately in two orthogonal planes [95]. Although similar to the division mode of *S. aureus*, *D. radiodurans* begins a second round of septation before the cells fully separate. Thus, in each hemispherical daughter cell, septation initiates from the center of the previous septum and from the middle of the cell periphery, resulting in a division plane that is orthogonal to the existing division septum [96]. This process, known as the ‘closing door mechanism’, can sometimes result in one side of the closing septum growing earlier or more rapidly than the other, causing asynchrony in septation [24]. As seen in *S. aureus*, *D. radiodurans* also undergoes peripheral cell wall synthesis in addition to septal cell wall synthesis during cell division [24]. Although the role of the Min system in *D. radiodurans* has not been studied, DivIVA is reportedly an essential protein in *D. radiodurans* and mutations in its C-terminal domain resulted in tilted septa, suggesting an involvement in the selection of the division plane [97,98]. In *D. radiodurans*, DivIVA interacts with MinC and components of the chromosome segregation machinery, indicating that it could play an important role in co-ordinating division and chromosome segregation [99]. Additionally, the phosphorylation of DivIVA has been shown to influence its function and dynamic localization during the *D. radiodurans* cell cycle [98]. Interestingly, the genome of *D. radiodurans* consists of four distinct replicons – two chromosomes and two plasmids – which can exist in multiple copies within the cells [24]. A detailed analysis of the nucleoid structure revealed that it is highly condensed, often adopting a toroidal shape [24] (Figure 4A). Examination of chromosome 1 indicated a greater number of *oriC* sites compared with *ter* sites. The chromosome exhibits a radial arrangement within the cell, with *oriC* sites evenly distributed around centrally located *ter* sites. Notably, the *ter* sites remained clustered in the central region of the cell until just before cytokinesis, although the responsible mechanism for this pattern remains unclear [24] (Figure 4A).

#### Neisseria gonorrhoeae

In the phylum Pseudomonadota (previously Proteobacteria), most cocci only encode the Min system, and SlmA is absent, indicating that the SlmA protein is restricted to *E. coli* and close relatives (Figure 2). The diplococcal pathogen *Neisseria gonorrhoeae* divides in alternating orthogonal planes, which overlap temporally, similar to the division pattern of *D. radiodurans* described above, and displays a subtle asymmetry that nonetheless establishes short and long axes [100,101]. Deletion of the *minCDE* operon resulted in severe morphological defects, disrupting Z-ring formation at mid-cell and resulting in minicell formation [100,102,103]. Surprisingly, when produced in round *E. coli* mutants, gonococcal Min proteins oscillated in a plane parallel to the existing septum, suggesting that the molecular mechanism of the Min system could be conserved in Pseudomonadota [104]. This suggests a model in which Min proteins oscillate along the long axis of ovoid cells, thereby restricting the division plane to a position perpendicular to the long axis (Figure 4B). Indeed, very recent work is consistent with this model and suggests co-ordination between cell division site selection and chromosome segregation [100].

## Concluding thoughts

The understanding of cell division in spherical bacteria has advanced rapidly over the past few years, perhaps due to increasing genetic manipulability of these organisms that has resulted in the establishment of new model systems to study bacterial cell division. Historically, Z-ring positioning in bacteria was thought to be broadly negatively regulated, as it is in *E. coli* by the Min system and nucleoid occlusion. However, recent research in Gram-positive cocci, such as *S. pneumoniae* and *S. aureus,* and the rod-shaped Gram-negative *Myxococcus xanthus* [105], highlight the importance of positive regulatory mechanisms in Z-ring placement in bacteria. This suggests that further studies are needed to uncover the mechanisms in other, less-studied bacteria that perhaps assume shapes that are neither cocci nor rods, where Z-ring positioning remains poorly understood.

Although spherical cells intrinsically lack cell polarity, recent studies reveal an emerging common theme wherein these cells may drive subtle morphological changes or overlap two rounds of the cell cycle to create a temporary long cellular axis that establishes ephemeral cell polarity. Interestingly, consecutive orthogonal cell division events appear to be common in spherical bacteria that are not ovoid in shape, suggesting potential advantages to this mode of cell division when the bacterium grows in its environmental niche or during pathogenesis. For example, the secretion of various *S. aureus* adhesins that are critical for host interaction at the division septum ensures the even distribution of these proteins across the cell surface after multiple rounds of cell division [106], presumably to permit these bacteria to better interact with their environment. Similarly, clustered growth, which naturally results from consecutive orthogonal cell division, may result in increased fitness in ecological niches in ways that are not readily apparent from growing cells in laboratory media [81]. Thus, although cell division in rod-shaped bacteria is typically thought of simply in terms of correctly dividing a parent cell to produce two nearly identical daughter cells, cell division in cocci may emerge as a more complex phenomenon that may permits groups of cells to thrive together in specific environmental conditions.

**Perspectives**

- Cocci utilize permanent or transient morphological changes to accurately position to the cell division machinery at mid-cell.
- Studying known mechanisms in understudied cocci could inform on variations in the mode of action of conserved cell division proteins.
- The conservation of Z-ring positioning mechanisms in cocci varies across groups and some species lack known mechanisms, suggesting that additional, yet-to-be-discovered mechanisms may exist.

## Supplementary material

online supplementary table 1

## Abbreviations

SBS: SlmA-binding sites
SCCs: serotype C carbohydrates
WTAs: wall teichoic acids

## References

[1] Yang X., Lyu Z., Miguel A., McQuillen R., Huang K.C., Xiao J (2017). GTPase activity-coupled treadmilling of the bacterial tubulin FtsZ organizes septal cell wall synthesis. Science.

[2] Bisson-Filho A.W., Hsu Y.P., Squyres G.R., Kuru E., Wu F., Jukes C. (2017). Treadmilling by FtsZ filaments drives peptidoglycan synthesis and bacterial cell division. Science.

[3] Mahone C.R., Goley E.D (2020). Bacterial cell division at a glance. J. Cell. Sci..

[4] Boer P.A., Crossley R.E., Rothfield L.I (1989). A division inhibitor and a topological specificity factor coded for by the minicell locus determine proper placement of the division septum in *E. coli*. Cell.

[5] Levin P.A., Margolis P.S., Setlow P., Losick R., Sun D (1992). Identification of *Bacillus subtilis* genes for septum placement and shape determination. J. Bacteriol..

[6] Rowlett V.W., Margolin W (2013). The bacterial min system. Curr. Biol..

[7] Ortiz C., Natale P., Cueto L., Vicente M (2016). The keepers of the ring: regulators of FtsZ assembly. FEMS Microbiol. Rev..

[8] Loose M., Fischer-Friedrich E., Herold C., Kruse K., Schwille P (2011). Min protein patterns emerge from rapid rebinding and membrane interaction of MinE. Nat. Struct. Mol. Biol..

[9] Patrick J.E., Kearns D.B (2008). MinJ (YvjD) is a topological determinant of cell division in *Bacillus subtilis*. Mol. Microbiol..

[10] Bramkamp M., Emmins R., Weston L., Donovan C., Daniel R.A., Errington J (2008). A novel component of the division‐site selection system of *Bacillus subtilis* and a new mode of action for the division inhibitor MinCD. Mol. Microbiol..

[11] Ramamurthi K.S., Losick R (2009). Negative membrane curvature as a cue for subcellular localization of a bacterial protein. Proc. Natl. Acad. Sci. U.S.A..

[12] Lenarcic R., Halbedel S., Visser L., Shaw M., Wu L.J., Errington J. (2009). Localisation of DivIVA by targeting to negatively curved membranes. EMBO J..

[13] Gregory J.A., Becker E.C., Pogliano K (2008). *Bacillus subtilis* MinC destabilizes FtsZ-rings at new cell poles and contributes to the timing of cell division. Genes Dev..

[14] Eswaramoorthy P., Erb M.L., Gregory J.A., Silverman J., Pogliano K., Pogliano J. (2011). Cellular architecture mediates DivIVA ultrastructure and regulates min activity in *Bacillus subtilis*. MBio.

[15] Feddersen H., Würthner L., Frey E., Bramkamp M (2021). Dynamics of the *Bacillus subtilis* min system. MBio.

[16] Bernhardt T.G., Boer P.A.J (2005). SlmA, a nucleoid-associated, FtsZ binding protein required for blocking septal ring assembly over chromosomes in *E. coli*. Mol. Cell.

[17] Tonthat N.K., Arold S.T., Pickering B.F., Van Dyke M.W., Liang S., Lu Y. (2011). Molecular mechanism by which the nucleoid occlusion factor, SlmA, keeps cytokinesis in check. EMBO J..

[18] Cho H., McManus H.R., Dove S.L., Bernhardt T.G (2011). Nucleoid occlusion factor SlmA is a DNA-activated FtsZ polymerization antagonist. Proc. Natl. Acad. Sci. U.S.A.

[19] Schumacher M.A., Zeng W (2016). Structures of the nucleoid occlusion protein SlmA bound to DNA and the C-terminal domain of the cytoskeletal protein FtsZ. Proc. Natl. Acad. Sci. U.S.A.

[20] Cabré E.J., Monterroso B., Alfonso C., Sánchez-Gorostiaga A., Reija B., Jiménez M. The nucleoid occlusion SlmA protein accelerates the disassembly of the FtsZ protein polymers without affecting their GTPase activity. PLoS ONE.

[21] Monterroso B., Zorrilla S., Sobrinos-Sanguino M., Robles-Ramos M.A., López-Álvarez M., Margolin W. (2019). Bacterial FtsZ protein forms phase-separated condensates with its nucleoid-associated inhibitor SlmA. EMBO Rep..

[22] Sievers J., Raether B., Perego M., Errington J (2002). Characterization of the parB-like yyaA gene of *Bacillus subtilis*. J. Bacteriol..

[23] Wu L.J., Errington J (2004). Coordination of cell division and chromosome segregation by a nucleoid occlusion protein in *Bacillus subtilis*. Cell.

[24] Wu L.J., Ishikawa S., Kawai Y., Oshima T., Ogasawara N., Errington J (2009). Noc protein binds to specific DNA sequences to coordinate cell division with chromosome segregation. EMBO J..

[25] Adams D.W., Wu L.J., Errington J (2015). Nucleoid occlusion protein Noc recruits DNA to the bacterial cell membrane. EMBO J..

[26] Jalal A.S.B., Tran N.T., Stevenson C.E., Chan E.W., Lo R., Tan X. (2020). Diversification of DNA-binding specificity by permissive and specificity-switching mutations in the ParB/Noc protein family. Cell Rep..

[27] Jalal A.S.B., Tran N.T., Wu L.J., Ramakrishnan K., Rejzek M., Gobbato G. (2021). CTP regulates membrane-binding activity of the nucleoid occlusion protein Noc. Mol. Cell.

[28] Eswara P.J., Ramamurthi K.S (2017). Bacterial cell division: nonmodels poised to take the spotlight. Annu. Rev. Microbiol..

[29] Young K.D (2006). The selective value of bacterial shape. Microbiol. Mol. Biol. Rev..

[30] Pinho M.G., Kjos M., Veening J.W (2013). How to get (a)round: mechanisms controlling growth and division of coccoid bacteria. Nat. Rev. Microbiol..

[31] Yulo P.R.J., Hendrickson H.L (2019). The evolution of spherical cell shape; progress and perspective. Biochem. Soc. Trans..

[32] Perez A.J., Boersma M.J., Bruce K.E., Lamanna M.M., Shaw S.L., Tsui H.C.T. (2021). Organization of peptidoglycan synthesis in nodes and separate rings at different stages of cell division of *Streptococcus pneumoniae*. Mol. Microbiol..

[33] Trouve J., Zapun A., Arthaud C., Durmort C., Di Guilmi A.M., Söderström B (2021). Nanoscale dynamics of peptidoglycan assembly during the cell cycle of *Streptococcus pneumoniae*. Curr. Biol..

[34] Perez A.J., Cesbron Y., Shaw S.L., Bazan Villicana J., Tsui H.C.T., Boersma M.J. (2019). Movement dynamics of divisome proteins and PBP2x:FtsW in cells of *Streptococcus pneumoniae*. Proc. Natl. Acad. Sci. U.S.A..

[35] Tsui H.C.T., Boersma M.J., Vella S.A., Kocaoglu O., Kuru E., Peceny J.K. (2014). Pbp2x localizes separately from Pbp2b and other peptidoglycan synthesis proteins during later stages of cell division of *Streptococcus pneumoniae* D39. Mol. Microbiol..

[36] Perez A.J., Lamanna M.M., Bruce K.E., Touraev M.A., Page J.E., Shaw S.L. (2024). Elongasome core proteins and class A PBP1a display zonal, processive movement at the midcell of *Streptococcus pneumoniae*. Proc. Natl. Acad. Sci. U.S.A..

[37] Straume D., Piechowiak K.W., Olsen S., Stamsås G.A., Berg K.H., Kjos M. (2020). Class A PBPs have a distinct and unique role in the construction of the pneumococcal cell wall. Proc. Natl. Acad. Sci. U.S.A..

[38] Paik J., Kern I., Lurz R., Hakenbeck R (1999). Mutational analysis of the *Streptococcus pneumoniae* bimodular class A penicillin-binding proteins. J. Bacteriol..

[39] Fenton A.K., Manuse S., Flores-Kim J., Garcia P.S., Mercy C., Grangeasse C. (2018). Phosphorylation-dependent activation of the cell wall synthase PBP2a in *Streptococcus pneumoniae* by MacP. Proc. Natl. Acad. Sci. U.S.A.

[40] Fleurie A., Lesterlin C., Manuse S., Zhao C., Cluzel C., Lavergne J.P. (2014). MapZ marks the division sites and positions FtsZ rings in *Streptococcus pneumoniae*. Nature.

[41] Holečková N., Doubravová L., Massidda O., Molle V., Buriánková K., Benada O. (2014). LocZ is a new cell division protein involved in proper septum placement in *Streptococcus pneumoniae*. MBio.

[42] Manuse S., Jean N.L., Guinot M., Lavergne J.P., Laguri C., Bougault C.M. (2016). Structure-function analysis of the extracellular domain of the pneumococcal cell division site positioning protein MapZ. Nat. Commun..

[43] Hosek T., Bougault C.M., Lavergne J.P., Martinez D., Ayala I., Fenel D. (2020). Structural features of the interaction of MapZ with FtsZ and membranes in *Streptococcus pneumoniae*. Sci. Rep..

[44] Zamakhaeva S., Chaton C.T., Rush J.S., Ajay Castro S., Kenner C.W., Yarawsky A.E. (2021). Modification of cell wall polysaccharide guides cell division in *Streptococcus mutans*. Nat. Chem. Biol..

[45] Flores-Kim J., Dobihal G.S., Bernhardt T.G., Rudner D.Z (2022). WhyD tailors surface polymers to prevent premature bacteriolysis and direct cell elongation in *Streptococcus pneumoniae*. Elife.

[46] Manuse S., Fleurie A., Zucchini L., Lesterlin C., Grangeasse C (2016). Role of eukaryotic-like serine/threonine kinases in bacterial cell division and morphogenesis. FEMS Microbiol. Rev..

[47] Beilharz K., Nováková L., Fadda D., Branny P., Massidda O., Veening J.W (2012). Control of cell division in *Streptococcus pneumoniae* by the conserved Ser/Thr protein kinase StkP. Proc. Natl. Acad. Sci. U.S.A..

[48] Trouve J., Zapun A., Bellard L., Juillot D., Pelletier A., Freton C. (2024). DivIVA controls the dynamics of septum splitting and cell elongation in *Streptococcus pneumoniae*. MBio.

[49] Fleurie A., Manuse S., Zhao C., Campo N., Cluzel C., Lavergne J.P. (2014). Interplay of the serine/threonine-kinase StkP and the paralogs DivIVA and GpsB in pneumococcal cell elongation and division. PLoS Genet..

[50] Fadda D., Santona A., D’Ulisse V., Ghelardini P., Ennas M.G., Whalen M.B. (2007). *Streptococcus pneumoniae* DivIVA: localization and interactions in a MinCD-free context. J. Bacteriol..

[51] Fleurie A., Cluzel C., Guiral S., Freton C., Galisson F., Zanella-Cleon I. (2012). Mutational dissection of the S/T-kinase StkP reveals crucial roles in cell division of *Streptococcus pneumoniae*. Mol. Microbiol..

[52] Jiang Q., Li B., Zhang L., Li T., Hu Q., Li H. (2023). DivIVA interacts with the cell wall hydrolase MltG to regulate peptidoglycan synthesis in *Streptococcus suis*. Microbiol. Spectr..

[53] Raaphorst R., Kjos M., Veening J.W (2017). Chromosome segregation drives division site selection in *Streptococcus pneumoniae*. Proc. Natl. Acad. Sci. U.S.A..

[54] Gallay C., Sanselicio S., Anderson M.E., Soh Y.M., Liu X., Stamsås G.A. CcrZ is a pneumococcal spatiotemporal cell cycle regulator that interacts with FtsZ and controls DNA replication by modulating the activity of DnaA. Nat. Microbiol..

[55] Yother J (2011). Capsules of *Streptococcus pneumoniae* and other bacteria: paradigms for polysaccharide biosynthesis and regulation. Annu. Rev. Microbiol..

[56] Mercy C., Ducret A., Slager J., Lavergne J.P., Freton C., Nagarajan S.N. (2019). RocS drives chromosome segregation and nucleoid protection in *Streptococcus pneumoniae*. Nat. Microbiol..

[57] Garcia P.S., Simorre J.P., Brochier-Armanet C., Grangeasse C (2016). Cell division of *Streptococcus pneumoniae*: think positive!. Curr. Opin. Microbiol..

[58] Rasmussen M (2013). Aerococci and aerococcal infections. J. Infect..

[59] Pérez-Núñez D., Briandet R., David B., Gautier C., Renault P., Hallet B. (2011). A new morphogenesis pathway in bacteria: unbalanced activity of cell wall synthesis machineries leads to coccus-to-rod transition and filamentation in ovococci. Mol. Microbiol..

[60] Tzagoloff H., Novick R (1977). Geometry of cell division in *Staphylococcus aureus*. J. Bacteriol..

[61] Saraiva B.M., Sorg M., Pereira A.R., Ferreira M.J., Caulat L.C., Reichmann N.T. (2020). Reassessment of the distinctive geometry of *Staphylococcus aureus* cell division. Nat. Commun..

[62] Wacnik K., Rao V.A., Chen X., Lafage L., Pazos M., Booth S. (2022). Penicillin-binding protein 1 (PBP1) of *Staphylococcus aureus* has multiple essential functions in cell division. MBio.

[63] Barbuti M.D., Myrbråten I.S., Morales Angeles D., Kjos M (2023). The cell cycle of *Staphylococcus aureus*: an updated review. Microbiologyopen.

[64] Monteiro J.M., Pereira A.R., Reichmann N.T., Saraiva B.M., Fernandes P.B., Veiga H. (2018). Peptidoglycan synthesis drives an FtsZ-treadmilling-independent step of cytokinesis. Nature.

[65] Schäper S., Brito A.D., Saraiva B.M., Squyres G.R., Holmes M.J., Garner E.C. (2024). Cell constriction requires processive septal peptidoglycan synthase movement independent of FtsZ treadmilling in *Staphylococcus aureus*. Nat. Microbiol..

[66] Zhou X., Halladin D.K., Rojas E.R., Koslover E.F., Lee T.K., Huang K.C. (2015). Bacterial division. Mechanical crack propagation drives millisecond daughter cell separation in *Staphylococcus aureus*. Science.

[67] Monteiro J.M., Fernandes P.B., Vaz F., Pereira A.R., Tavares A.C., Ferreira M.T. (2015). Cell shape dynamics during the staphylococcal cell cycle. Nat. Commun..

[68] Reichmann N.T., Tavares A.C., Saraiva B.M., Jousselin A., Reed P., Pereira A.R. (2019). SEDS-bPBP pairs direct lateral and septal peptidoglycan synthesis in *Staphylococcus aureus*. Nat. Microbiol..

[69] Sutton J.A.F., Cooke M., Tinajero-Trejo M., Wacnik K., Salamaga B., Portman-Ross C. The roles of GpsB and DivIVA in *Staphylococcus aureus* growth and division. Front. Microbiol..

[70] Costa S.F., Saraiva B.M., Veiga H., Marques L.B., Schäper S., Sporniak M. (2024). The role of GpsB in *Staphylococcus aureus* cell morphogenesis. MBio.

[71] Eswara P.J., Brzozowski R.S., Viola M.G., Graham G., Spanoudis C., Trebino C. (2018). An essential *Staphylococcus aureus* cell division protein directly regulates FtsZ dynamics. Elife.

[72] Hammond L.R., Sacco M.D., Khan S.J., Spanoudis C., Hough-Neidig A., Chen Y. (2022). GpsB coordinates cell division and cell surface decoration by wall teichoic acids in *Staphylococcus aureus*. Microbiol. Spectr..

[73] Sacco M.D., Hammond L.R., Noor R.E., Bhattacharya D., McKnight L.J., Madsen J.J. (2024). *Staphylococcus aureus* FtsZ and PBP4 bind to the conformationally dynamic N-terminal domain of GpsB. Elife.

[74] Bartlett T.M., Sisley T.A., Mychack A., Walker S., Baker R.W., Rudner D.Z. (2024). FacZ is a GpsB-interacting protein that prevents aberrant division-site placement in *Staphylococcus aureus*. Nat. Microbiol..

[75] Anderson J.S., Meadow P.M., Haskin M.A., Strominger J.L (1966). Biosynthesis of the peptidoglycan of bacterial cell walls. I. Utilization of uridine diphosphate acetylmuramyl pentapeptide and uridine diphosphate acetylglucosamine for peptidoglycan synthesis by particulate enzymes from *Staphylococcus aureus* and Micrococcus lysodeikticus. Arch. Biochem. Biophys..

[76] Turner R.D., Ratcliffe E.C., Wheeler R., Golestanian R., Hobbs J.K., Foster S.J Peptidoglycan architecture can specify division planes in *Staphylococcus aureus*. Nat. Commun..

[77] Iwaya M., Goldman R., Tipper D.J., Feingold B., Strominger J.L (1978). Morphology of an *Escherichia coli* mutant with a temperature-dependent round cell shape. J. Bacteriol..

[78] Begg K.J., Donachie W.D (1998). Division planes alternate in spherical cells of *Escherichia coli*. J. Bacteriol..

[79] Veiga H., Jorge A.M., Pinho M.G (2011). Absence of nucleoid occlusion effector Noc impairs formation of orthogonal FtsZ rings during *Staphylococcus aureus* cell division. Mol. Microbiol..

[80] Pang T., Wang X., Lim H.C., Bernhardt T.G., Rudner D.Z (2017). The nucleoid occlusion factor Noc controls DNA replication initiation in *Staphylococcus aureus*. PLoS Genet..

[81] Ramos-León F., Anjuwon-Foster B.R., Anantharaman V., Updegrove T.B., Ferreira C.N., Ibrahim A.M. (2024). PcdA promotes orthogonal division plane selection in *Staphylococcus aureus*. Nat. Microbiol..

[82] Bottomley A.L., Liew A.T.F., Kusuma K.D., Peterson E., Seidel L., Foster S.J. Coordination of chromosome segregation and cell division in *Staphylococcus aureus*. Front. Microbiol..

[83] Holt S.C., Canale-Parola E (1967). Fine structure of *Sarcina maxima* and *Sarcina ventriculi*. J. Bacteriol..

[84] Hammond L.R., White M.L., Eswara P.J (2019). ¡vIVA la DivIVA!. J. Bacteriol..

[85] Willemse J., Borst J.W., Waal E., Bisseling T., Wezel G.P (2011). Positive control of cell division: FtsZ is recruited by SsgB during sporulation of Streptomyces. Genes Dev..

[86] Ramos-León F., Bush M.J., Sallmen J.W., Chandra G., Richardson J., Findlay K.C. (2021). A conserved cell division protein directly regulates FtsZ dynamics in filamentous and unicellular actinobacteria. Elife.

[87] Miyagishima S.Y., Wolk C.P., Osteryoung K.W (2005). Identification of cyanobacterial cell division genes by comparative and mutational analyses. Mol. Microbiol..

[88] Springstein B.L., Nürnberg D.J., Weiss G.L., Pilhofer M., Stucken K Structural determinants and their role in cyanobacterial morphogenesis. Life.

[89] Mazouni K., Domain F., Cassier‐Chauvat C., Chauvat F (2004). Molecular analysis of the key cytokinetic components of cyanobacteria: FtsZ, ZipN and MinCDE. Mol. Microbiol..

[90] Marbouty M., Saguez C., Cassier-Chauvat C., Chauvat F (2009). ZipN, an FtsA-like orchestrator of divisome assembly in the model cyanobacterium *Synechocystis* PCC6803. Mol. Microbiol..

[91] MacCready J.S., Schossau J., Osteryoung K.W., Ducat D.C (2017). Robust Min-system oscillation in the presence of internal photosynthetic membranes in cyanobacteria. Mol. Microbiol..

[92] Bharat T.A.M., Tocheva E.I., Alva V (2023). The cell envelope architecture of *Deinococcus*: HPI forms the S-layer and SlpA tethers the outer membrane to peptidoglycan. Proc. Natl. Acad. Sci. U.S.A..

[93] Farci D., Haniewicz P., Piano D (2022). The structured organization of *Deinococcus radiodurans*’ cell envelope. Proc. Natl. Acad. Sci. U.S.A..

[94] Farci D., Piano D (2023). Reply to Bharat et al.: continuity or discontinuity, that is the question. Proc. Natl. Acad. Sci. U.S.A..

[95] Murray R.G.E., Hall M., Thompson B.G (1983). Cell division in *Deinococcus* radiodurans and a method for displaying septa. Can. J. Microbiol..

[96] Floc’h K., Lacroix F., Servant P., Wong Y.S., Kleman J.P., Bourgeois D. (2019). Cell morphology and nucleoid dynamics in dividing *Deinococcus* radiodurans. Nat. Commun..

[97] Chaudhary R., Kota S., Misra H.S (2021). DivIVA regulates its expression and the orientation of new septum growth in *Deinococcus* radiodurans. J. Bacteriol..

[98] Chaudhary R., Kota S., Misra H.S (2023). DivIVA phosphorylation affects its dynamics and cell cycle in radioresistant *Deinococcus* radiodurans. Microbiol. Spectr..

[99] Chaudhary R., Gupta A., Kota S., Misra H.S (2019). N-terminal domain of DivIVA contributes to its dimerization and interaction with genome segregation proteins in a radioresistant bacterium *Deinococcus* radiodurans. Int. J. Biol. Macromol..

[100] Bandekar A.C., Ramirez-Diaz D.A., Palace S.G., Wang Y., Garner E.C., Grad Y.H Axial asymmetry organizes division plane orthogonality in *Neisseria gonorrhoeae*. bioRxiv.

[101] Westling-Häggström B., Elmros T., Normark S., Winblad B (1977). Growth pattern and cell division in *Neisseria gonorrhoeae*. J. Bacteriol..

[102] Victor C., Dillon J.A.R., Francis F., Szeto J., Beveridge T.J., Ramirez-Arcos S (2001). Deletion of the cell-division inhibitor MinC results in lysis of *Neisseria gonorrhoeae*. Microbiology.

[103] Parti R.P., Horbay M.A., Liao M., Dillon J.A.R (2013). Regulation of minD by oxyR in *Neisseria gonorrhoeae*. Res. Microbiol..

[104] Ramirez‐Arcos S., Szeto J., Dillon J.R., Margolin W (2002). Conservation of dynamic localization among MinD and MinE orthologues: oscillation of *Neisseria gonorrhoeae* proteins in *Escherichia coli*. Mol. Microbiol..

[105] Treuner-Lange A., Aguiluz K., Does C., Gómez-Santos N., Harms A., Schumacher D (2013). PomZ, a ParA-like protein, regulates Z-ring formation and cell division in *Myxococcus xanthus*. Mol. Microbiol..

[106] Schneewind O., Missiakas D.M (2019). Staphylococcal protein secretion and envelope assembly. Microbiol. Spectr..

