# Supplementary material for: How do spherical bacteria regulate cell division?
Source: Biochem Soc Trans. 2025 Apr 17;53(2):447–60. doi: 10.1042/BST20240956 (PMC12203958; doi:10.1042/BST20240956)
Supplement: online supplementary table 1 [file BST-53-02-BST20240956-s001.docx]

**Supplementary Table 1. Conserved proteins involved in Z-ring positioning across different bacterial species.** Homologs were identified through BLAST searches using sequences from the following well-characterized proteins (locus tags in parentheses): Min system proteins from *Escherichia coli* K-12 MG1655—MinC (b1176), MinD (b1175), and MinE (b1174); MinJ from *Bacillus subtilis* 168 (BSU_35220); DivIVA from *B. subtilis* 168 (BSU_15420); PcdA from *Staphylococcus aureus* USA300 FPR3757 (SAUSA300_2094); MapZ from *Streptococcus pneumoniae* sv 2 D39 (SPD_0342); Noc from *B. subtilis* 168 (BSU_40990); Cdv3, a cyanobacterial DivIVA paralog, from *Synechocystis* sp. PCC 6803 (Sll0848); and SlmA from *E. coli* K-12 MG1655 (b3641). The percentage identity of each homolog to its corresponding reference protein is indicated in red below the locus tag. An asterisk next to cyanobacterial DivIVA proteins denotes the paralog Cdv3. Genomes used for each species are listed in the legend of Figure 4. MinJ and SlmA are not present in the table since they are restricted to *B. subtilis* and *E. coli* respectively.

|  | MinC | MinD | MinE | DivIVA | PcdA | MapZ | Noc |
| --- | --- | --- | --- | --- | --- | --- | --- |
| *Aerococcus urinae* CCUG 36881 |  |  |  | AWM73_02870  27% |  |  |  |
| *Deinococcus radiodurans* R1 | DR_1753  38% | DR_0752  49% | DR_0751  30% | DR_1369*  26% |  |  |  |
| *Enterococcus faecalis* Portland ATCC 29212 |  |  |  | DR75_62  35% |  | DR75_529  46% |  |
| *Gemella haemolysans* NCTC 10459 |  |  |  |  |  |  | NCTC10459_00185  44% |
| *Gloeocapsa* sp. PCC 7428 | Glo7428_4847  31% | Glo7428_4848  46% | Glo7428_4849  39% | Glo7428_0003*  32% |  |  |  |
| *Lactococcus lactis lactis* IL1403 |  |  |  | L117685  33% |  | L98109  40% |  |
| *Macrococcus caseolyticus* JCSC5402 |  |  |  | MCCL_0763  42% | MCCL_1808  32% |  | MCCL_1952  50% |
| *Mammaliicoccus sciuri* FDAARGOS_285 |  |  |  | CEP64_07605  42% | CEP64_02550  56% |  | CEP64_12490  50% |
| *Methylococcus capsulatus* Bath | MCA3066  40% | MCA3067  73% | MCA3068  50% |  |  |  |  |
| *Micrococcus luteus* NCTC 2665 |  |  |  | Mlut_13520  25% |  |  |  |
| *Moraxella catarrhalis* BBH18 | MCR_1598  44% | MCR_1599  64% | MCR_1600  32% |  |  |  |  |
| *Neisseria gonorrhoeae* FA 1090 | NGO_1816  36% | NGO_09675  75% | NGO_1814  42% |  |  |  |  |
| *Nosocomiicoccus ampullae* DSM 19163 |  |  |  |  |  |  | KPF49_07990  45% |
| *Prochlorococcus marinus* MIT9313 | PMT_1652  34% | PMT_1653  42% | PMT_1654  46% | PMT_0721*  24% |  |  |  |
| *Salinicoccus halodurans* H3B36 |  |  |  | AAT16_05460  42% |  |  | AAT16_00155  46% |
| *Sarcina ventriculi* NCTC 12966 | NCTC12966_01790  37% | NCTC12966_01791  48% | NCTC12966_01792  27% | NCTC12966_01479  31% |  |  |  |
| *Staphylococcus aureus* USA300 FPR3757 |  |  |  | SAUSA300_1086  41% | SAUSA300_2094  100% |  | SAUSA300_2643  48% |
| *Streptococcus pneumoniae* sv 2 D39 |  |  |  | SPD_1474  37% |  | SPD_0342  100% |  |
| *Synechocystis* sp. PCC 6803 | Sll0288  31% | Sll0289  45% | Ssl0546  34% | Sll0848*  100% |  |  |  |
| *Veillonella parvula* DSM 2008 |  |  |  |  |  |  |  |
